# Präimplementierung von elektronischen Patientenberichten an Referenzzentren für Kopf-Hals-Onkologie: Ein Fahrplan in Richtung patientenzentrierter Digitalisierung

**DOI:** 10.1007/s00106-024-01543-7

**Published:** 2025-01-16

**Authors:** Daniel Dejaco, Timo Gottfried, Matthias Santer, Anna Thurner, Jens Lehmann, David Riedl, Gerhard Rumpold, Bernhard Holzner, Joachim Schmutzhard, Benedikt Hofauer

**Affiliations:** 1 Universitätsklinik für Hals‑, Nasen- und Ohrenheilkunde, Kopf- und Halschirurgie Innsbruck, Medizinische Universität Innsbruck, Anichstr. 35, 6020 Innsbruck, Österreich; 2 Department für Psychiatrie, Psychotherapie, Psychosomatik und Medizinische Psychologie Innsbruck, Universitätsklinik für Psychiatrie II Innsbruck, Medizinische Universität Innsbruck, Schöpfstr. 23a, 6020 Innsbruck, Österreich; 3 Evaluation Software Development, Valiergasse 62, 6020 Innsbruck, Österreich

**Keywords:** Lebensqualität, Erhebungen und Fragebögen, Computergestützte Signalverarbeitung, Elektronische Datenverarbeitung, Datenbankenverwaltungssysteme, Quality of life, Surveys and questionnaires, Computer-assisted signal processing, Electronic data processing, Database management systems

## Abstract

**Hintergrund:**

Elektronisch erfasste Patientenberichte („electronic patient-reported outcomes“, ePRO) sind digitale, von Krebspatienten ausgefüllte Fragebögen. Trotz Hinweisen auf eine Verbesserung der klinischen Versorgung, ist die Integration von ePRO in der klinischen Kopf-Hals-Onkologie Neuland.

**Ziel der Arbeit:**

Diese Arbeit skizziert den Implementierungsprozess für ePRO der Universitätsklinik für Hals‑, Nasen- und Ohrenheilkunde der Medizinischen Universität Innsbruck (HNO Innsbruck).

**Methoden:**

Die Implementierung erfolgt durch eine Projektteam in einer Präimplementierungsphase (Bedarfsevaluation, Implementierungsplanung, Identifikation innerklinischer Barrieren, Prototypenentwicklung, Testung und Adaptierung sowie Anwenderschulung), Implementierungsphase (Implementierung und Anwendertraining) und Postimplementierungsphase (Qualitätskontrolle, Projektausweitung).

**Ergebnisse:**

Das Projektteam an der HNO Innsbruck besteht aus 10 Mitgliedern, die Digitalisierungsbedarf in der Krebsnachsorge identifizierten. Eine Hybridimplementierunglösung („Computer-based Health Evaluation System“, CHES; Fa. Evaluation Software Development, ESD, Innsbruck, Österreich) wurde gewählt. ePRO („European Organization for Research and Treatment of Cancer Quality of Life Questionnaire – 30 items“, EORTC-QLQ-C30; Head and Neck Functional Integrity Scale, HNC-FIT Scale; und EORTC Head and Neck Cancer Module, EORTC H&N43) werden 12-mal über 5,5 Jahre erhoben. Insgesamt 25 Anwender bewerten den Prototypen als benutzerfreundlich (Patientensicht: 8,1 ± 1,6; 3–10; Anwendersicht: 8,6 ± 1,1; 6–10). Als Hauptvorteil wurde die schnellere Anamnese (72 %), als Hauptnachteil fehlendes Personal, Zeit und Motivation (52 %) genannt.

**Schlussfolgerung:**

Das Feedback zum ePRO-Prototypen in der HNO Innsbruck war positiv. Die Implementierungsphase wurde im ersten Quartal 2024 gestartet. Die Zielerreichung wird in der Postimplementierungsphase im vierten Quartal 2024 evaluiert.

## Abstract (en)

**Background:**

Electronically captured patient reports (electronic patient-reported outcomes, ePROs) are digital questionnaires filled out by cancer patients. Despite indications of improved clinical care, the integration of ePROs into clinical head and neck oncology is uncharted territory.

**Objective:**

This work outlines the implementation process for ePROs at the University Hospital for Otorhinolaryngology at the Medical University of Innsbruck (ENT Innsbruck).

**Methods:**

The implementation is carried out by a project team in a pre-implementation phase (needs assessment, implementation planning, identification of intra-hospital barriers, prototype development, testing and adaptation, and user training), an implementation phase (implementation and user training), and a post-implementation phase (quality control and project expansion).

**Results:**

The project team at ENT Innsbruck, consisting of 10 members, identified the need for digitalization in cancer follow-up. A hybrid implementation solution (Computer-Based Health Evaluation System, CHES; Evaluation Software Development, ESD, Innsbruck, Austria) was chosen. ePROs (European Organization for Research and Treatment of Cancer Quality of Life Questionnaire – 30 items, EORTC-QLQ-C30; Head and Neck Functional Integrity Scale, HNC-FIT scale; and EORTC Head and Neck Cancer Module, EORTC H&N43) will be collected 12 times over 5.5 years. A total of 25 users rated the prototype as user friendly (patient perspective: 8.1 ± 1.6, 3–10; user perspective: 8.6 ± 1.1, 6–10). The main advantage was faster medical history taking (72%), and the main disadvantages were a lack of personnel, time, and motivation (52%).

**Conclusion:**

The feedback on the ePRO prototype at ENT Innsbruck was positive. The implementation phase commenced in the first quarter of 2024. Goal achievement will be evaluated in the post-implementation phase in the fourth quarter of 2024.

## Vorteile elektronisch erfasster Patientenberichte

Elektronisch erfasste Patientenberichte (ePRO) sind digitale, von Krebspatienten ausgefüllte Fragebögen [1]. Sie fördern die fokussierte Anamnese, verbessern Therapiemanagement, Symptome, Lebensqualität und Funktion Betroffener [1–6]. Im Vergleich zu papierbasierten PRO wird die Komplettierung „zu Hause“ auch von älteren Patienten bevorzugt und die Datenqualität verbessert [7, 8]. ePRO reduzieren ungeplante Vorstellungen in ärztlicher Behandlung und Hospitalisierungszeiten [9], was Personal entlastet und Kosten senkt [7, 9].

Trotz dieser Vorteile [1–13] ist die klinische Implementierung begrenzt [14–16], wofür v.a. die Motivation von Klinikern und Patienten entscheidend ist [17, 18]. Die klinische Integration in der Kopf-Hals-Onkologie ist selten. Bisher gab es nur in Dänemark entsprechende Bestrebungen [19]. Weder in Deutschland, Österreich noch in der Schweiz wurde eine klinische Implementierung in der Kopf-Hals-Onkologie dokumentiert.

In der vorliegenden Arbeit wird der klinische ePRO-Implementierungsprozess für Referenzzentren für Kopf-Hals-Onkologie am Beispiel der Universitätsklinik für Hals‑, Nasen- und Ohrenheilkunde der Medizinischen Universität Innsbruck (HNO Innsbruck) unter Berücksichtigung von Empfehlungen des National Health Service (NHS) und der European Organization for Research and Treatment of Cancer (EORTC) [20, 21] skizziert.

## Material

### Phase 1: Präimplementierungsphase

Diese Phase wird durch ein Projektteam betreut und umfasst Bedarfsevaluation, Implementierungsplanung, Identifikation innerklinischer Barrieren, Prototypenentwicklung, Testung, Adaptierung und Anwenderschulung [20, 21].

#### Projektteam und klinische „Champions“

Das Projektteam besteht aus Ärzten, Pflege, Psychologen, IT-Technikern, Verwaltung und Patienten. In Meetings werden Aufgaben definiert und Bedürfnisse berücksichtigt [20, 21]. Ein klinischer „Champion“ wird definiert, der die Implementierung vorantreibt und als Bindeglied zwischen Projektteam und Anwendern fungiert [20, 21].

#### Bedarfsevaluation für ePRO

Hier unterscheidet man zwischen allgemeiner und spezieller Bedarfsevaluation. Erstere exploriert den ePRO-Bedarf, Letztere schätzt die Anzahl der Patienten mit Kopf-Hals-Karzinomen pro Jahr in der Therapie- und Nachsorgephase im ambulanten und stationären Setting [20, 21].

#### Implementierungsplanung und Identifikation innerklinischer Barrieren

Dabei werden Einschlusskriterien für Patienten und Behandlungsphasen, Erhebungszeitpunkte und -zeitrahmen, Identifikations- und Rekrutierungsprozess, technische Lösungen, die Fragebogenauswahl und finanzielle/personelle Umsetzbarkeit definiert [20, 21].

##### Formen der ePRO-Implementierung und Auswahlkriterien.

Es werden 3 Formen unterschieden: Bei der *vollständigen Implementierung* werden ePRO in das bestehende klinische Informationssystem (KIS) integriert. Dateneingabe und Analyse erfolgen im KIS. Diese Lösung ist oft unflexibel, auch wenn die Kosten im Vergleich zu kommerziellen Lösungen geringer sind. Schnittstellenprobleme können die Effektivität der Implementierung beeinträchtigen. Die *eigenständige Implementierung* nutzt kommerzielle Software als web- und/oder App-basiertes System. Dateneingabe und Analyse erfolgt auf externen Plattformen. Vorteile sind Flexibilität und effiziente Implementierung. Nachteile sind höhere Kosten und die Notwendigkeit zur Registrierung auf einer externen Plattform. Die *Hybridimplementierung* nutzt kommerzielle Software als web- und/oder App-basiertes System, ermöglicht aber die Datenanalyse im KIS mittels begrenzter Schnittstelle. Die Dateneingabe erfolgt weiterhin auf einer externen Plattform [20, 21].

Die Auswahl basiert auf technischer und finanzieller Realisierbarkeit. Benutzerfreundlichkeit hat oberste Priorität: Anwender sollten Daten im KIS abrufen können. Die Registrierung und Dateneingabe auf externen Plattformen sollten einfach gestaltet sein und an vertrauten Endgeräten möglich sein. Neue klinische Pfade sollten integrierbar sein, und Kliniker sollten neue Fragebögen ohne technischen Support ergänzen können. Patienten sollten automatisch an definierten Zeitpunkten an die Dateneingabe erinnert werden. Automatisch generierte Rückmeldungen an die Behandler beim Erreichen klinischer Cut-off-Werte sollten möglich sein [20, 21].

##### Auswahl der Fragebögen, Definition von Erfassungszeitpunkten und Schwellenwerten.

Bei der Fragebogenauswahl sollten die Anzahl, die Länge und die Erhebungszeitpunkte in Bezug auf den klinischen Mehrnutzen in Relation zur Belastung für Patienten mit Kopf-Hals-Karzinomen gesetzt werden. Die Fragebögen sollten verständlich, die Antwortkategorien klinisch relevant sein [20, 21].

Es gibt keine Empfehlungen zur Fragebogenauswahl in der klinischen Routine. In Studien wurden der EORTC-QLQ-C30 („European Organization for Research and Treatment of Cancer Quality of Life Questionnaire – 30 items“) [22], EORTC H&N43 (EORTC Head and Neck Cancer Module) [23] und die Head and Neck Functional Integrity Scale (HNC-FIT Scale) [24] verwendet. Für die Eigenschaften dieser Fragebögen wird auf die Literatur verwiesen [22–24]. Auch für die Erhebungszeitpunkte gibt es keine Empfehlungen für die klinische Routine. Während für die Nachsorge von Patient mit Kopf-Hals-Karzinomen gewisse Intervalle definiert sind (Jahr 1: 1–3 Monate, Jahr 2: 2–6 Monate, Jahr 3–5: 4–8 Monate und > Jahr 5: 12 Monate) [25], liegen keine Empfehlungen für die Therapiephase vor.

Abschließend sollte evaluiert werden, ob automatische Rückmeldungen an Kliniker generiert werden sollen. Dafür definiert das Projektteam Schwellenwerte, basierend auf etablierten klinischen Cut-off-Werten [26]. Eine Differenzierung zwischen Symptomen, die einer zeitnahen Behandlung bedürfen, und Symptomen, die bei geplanten Kontrollen exploriert werden können, wird empfohlen. Rückmeldungen können die Routineversorgung nicht ersetzen, sondern nur ergänzen. Obwohl langfristig eine Personalentlastung zu erwarten ist, werden kurzfristig zusätzliche Personalressourcen als Ansprechpartner nötig sein [20, 21].

#### Evaluation des ePRO-Prototypen

**Abb. 1:**
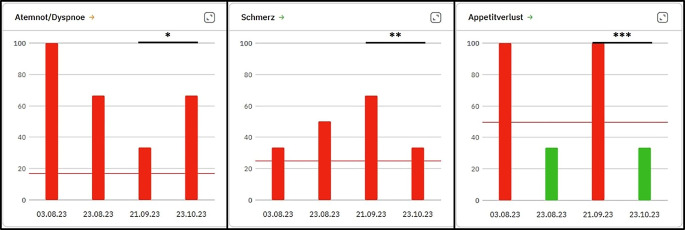
Exemplarische Darstellung von ePRO-Symptomdaten erhoben mittels EORTC-QLQ-C30 („European Organization for Research and Treatment of Cancer Quality of Life Questionnaire – 30 items“) [22] im „Computer-based Health Evaluation System“ (CHES) [27]. Exemplarische Darstellung von ePRO-Symptomdaten (EORTC-QLQ-C30 [22]) für Atemnot, Schmerz und Appetitverlust eines Dummy-Patienten in CHES [27]. Erläuterung s. Text. *Y‑Achse* Symptomausprägung, *x‑Achse *Untersuchungszeitpunkte. *Rote Balken *pathologische Symptomausprägungen, *grüne Balken *normale Symptomausprägungen (*rotbraune horizontale Linie *Referenzwerte) [26]

Nach Erstellung eines funktionsfähigen ePRO-Prototypen sollte der Prototyp von Anwendern getestet werden, um technische Probleme zu identifizieren und Feedback zu generieren [20, 21]. Für die Evaluation in der Pilotpopulation existieren keine Fragebögen. An der HNO Innsbruck wurden potenzielle Anwender in diese Studie eingeschlossen, ihr Alter, Geschlecht und Berufserfahrung erfasst, und sie wurden gebeten, den EORTC-QLQ-C30 [22] aus Patientensicht zu komplettieren. Im Anschluss wurden sie gebeten, Symptome und Funktion eines Dummy-Patienten, erhoben mittels EORTC-QLQ-C30 [22] und HNC-FIT Scale [24], zu identifizieren, die zuletzt pathologisch [26] waren, die sich verschlechtert bzw. verbessert hatten oder die zuletzt normal waren (Abb. 1). Die Interpretationszeit wurde erhoben. Die Anwender bewerteten dann die Benutzerfreundlichkeit aus Patienten- und Anwendersicht, ihre Erfahrung mit ePRO sowie den erwarteten Nutzen und Hürden auf einer Skala von 0 (schlechtester Wert) bis 10 (bester Wert). Gründe für die Bewertung wurden exploriert.

Wie in Abb. 1 dargestellt, wurden Anwender gebeten, Symptome zu identifizieren, die zum letzten Untersuchungszeitpunkt entweder pathologisch [26] waren oder die sich im Vergleich zur Voruntersuchung verschlechtert bzw. verbessert hatten oder die zum letzten Untersuchungszeitpunkt normal waren. Höhere Werte entsprechen einer stärkeren Symptomausprägung. Am 21.09. zeigten sich sowohl Atemnot/Dyspnoe, Schmerz als auch der Appetitverlust pathologisch. Während sich die Atemsituation am 23.10. weiter verschlechtert hatte, verbesserte sich die Schmerzsituation und der Appetitverlust erreichte sogar wieder Normwerte.

Die Auswertung erfolgte mit SPSS (Version 28, Fa. IBM Corp., Armonk/NY, USA) wobei die korrekten Interpretationen als Prozentwert richtiger Antworten und die Beurteilung auf den Skalen von 0 bis 10 numerisch dargestellt wurden. Mittelwerte, Standardabweichungen, Minimum und Maximum wurden berechnet, Anmerkungen tabellarisch kategorisiert.

#### Anwenderschulungen

Anwender sollten trainiert werden, ePRO-Daten vor Aufrufen der Patienten mit Kopf-Hals-Karzinomen zu überprüfen, relevante Symptome zu identifizieren und Änderungen im Verlauf zu erkennen. Kommunikationstrainings sollten Aspekte der Wertschätzung für die Fragebogenausfüllung und die Verbalisierung von wichtigen Problemen umfassen. Der Umgang mit besonders schwer belasteten Patienten sollte ebenfalls geschult werden. Hier fungiert der „klinische Champion“ als Bindeglied zwischen Anwendern und dem Projektteam als greifbarer Ansprechpartner und Treiber der Implementierung. Sorgen und Bedenken aller Berufsgruppen sollten ernst genommen werden, während gleichzeitig die Vorteile von ePRO betont werden sollten [20, 21].

### Phase 2: Implementierungsphase

Für diese Phase wird ein Starttermin definiert. Ein technischer Support für auftretende Probleme soll eingerichtet werden und alle beteiligten Berufsgruppen eine abschließendes Anwendertraining erhalten. In diesem können Prozessabläufe an „Dummy-Patienten“ trainiert werden. Die Einrichtung einer E‑Mail-Adresse für Fragen ist geplant. Das Projektteam wird in den ersten 3 Monaten neben dem „klinischen Champion“ zur vollen Verfügung stehen. Probleme werden gesammelt und 3 Monaten nach dem Starttermin in erneuten Anwendertrainings diskutiert werden [20, 21].

Die Ersteinführung für Patienten mit Kopf-Hals-Karzinomen soll durch Kliniker erfolgen, um ePRO als Bestandteil der Routine Versorgung zu etablieren. Schriftliches Informationsmaterial soll zur Verfügung gestellt werden. Es soll in verständlicher Sprache Information zur Speicherung, Zugang und Verwendung der Daten, klare Anleitungen zur Registrierung und Dateneingabe und Kontaktinformationen für technische Probleme beinhalten. Die Einwilligung zur Teilnahme erfolgt elektronisch bei der ersten Registrierung [20, 21].

### Phase 3: Postimplementierungsphase

Etwa 6 Monaten nach dem Starttermin beginnt diese Phase. Hier liegt der Fokus auf Qualitätskontrolle und der Ausweitung. Das Projektteam und der Champion führen Visiten durch, um sicherzustellen, dass Arbeitsabläufe eingehalten werden. Anwendertrainings v. a. für neues Personal sollten alle 3–6 Monate angeboten werden. Lösungen für dauerhaft funktionierenden Arbeitsabläufe sollten etabliert werden. Weitere Champions können etabliert und Stellvertreter für Schlüsselpositionen definiert werden. Vor der Auflösung des Projektteams sollten die gesammelten Daten analysiert und der Geschäftsführung und dem Qualitätsmanagement präsentiert werden [20, 21].

## Resultate

### Phase 1: Präimplementierungsphase

#### ePRO-Projektteams und „klinische Champions“

**Abb. 2:**
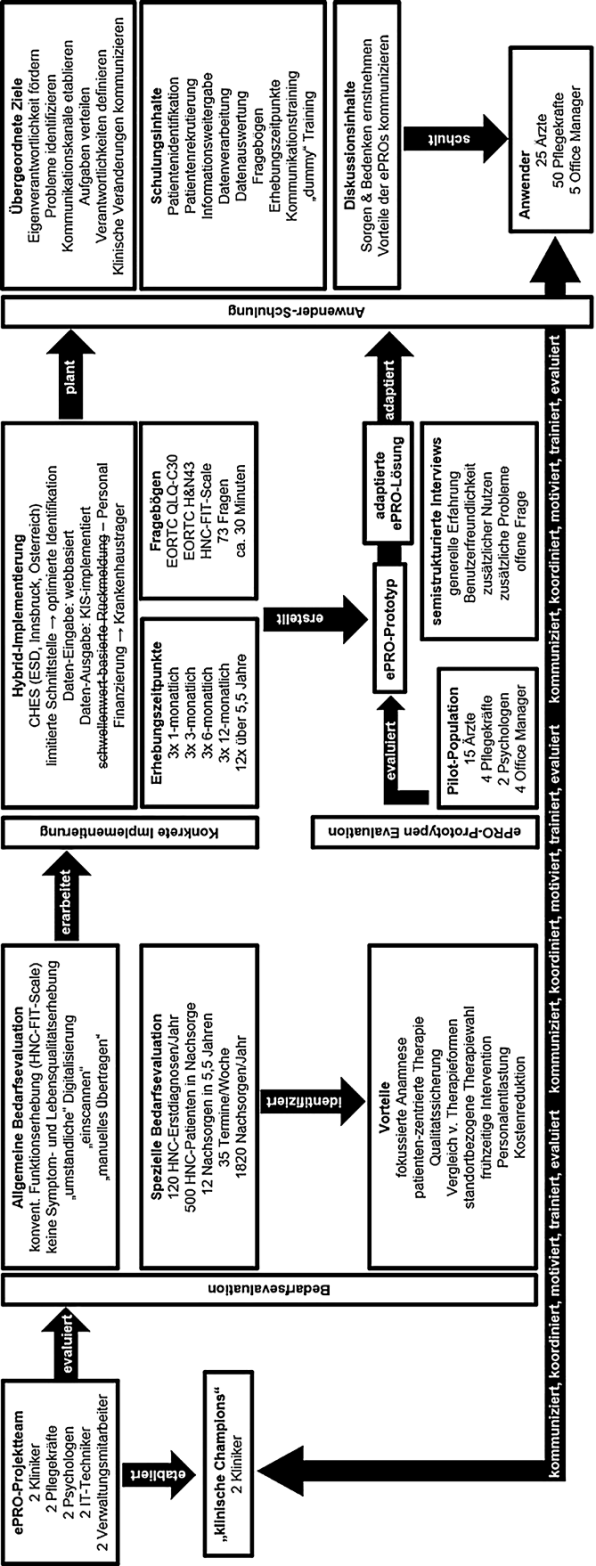
ePRO-Präimplementierungsphase an der HNO Innsbruck. Schematische Abbildung der ePRO-Präimplementierungsphase an der HNO Innsbruck als Flussdiagramm. Zusätzliche Details und Ergebnisse s. Text bzw. Tab. 1, Tab. 2, Tab. 3. *CHES *„Computer-based Health Evaluation System“, *ESD *Fa. Evaluation Software Development. *EORTC H&N43 *EORTC Head and Neck Cancer Module,* EORTC-QLQ-C30 *„European Organization for Research and Treatment of Cancer Quality of Life Questionnaire – 30 items“, *ePRO *elektronisch erfasste Patientenberichte („electronic patient-reported outcomes“), *HNC-FIT Scale *Head and Neck Functional Integrity Scale, *KIS *klinisches Informationssystem

An der HNO Innsbruck besteht das ePRO-Projektteam aus jeweils 2 Ärzten, Pflege, Psychologen, IT-Technikern und Verwaltung. An unserer Institution wurden 2 Champions (Fachärzte) etabliert (Abb. 2).

#### Bedarfsevaluation für ePRO

Funktionsniveaus werden an der HNO Innsbruck seit 2018 mittels HNC-FIT Scale [24] erfasst. Die Erhebung von Symptomen und Lebensqualität mittels EORTC-QLQ-C30 [22] und EORTC H&N43 [23] erfolgte bisher nur in Studien [24]. Diese Daten wurden papierbasiert erfasst, wobei die umständliche Digitalisierung als zentraler Nachteil identifiziert wurde. Digitalisierung könnten Therapieformen vergleichbarer und Daten für die Qualitätssicherung bzw. standortbezogene Therapieentscheidungen generieren. ePRO unterstützen frühzeitige Interventionen und könnten den Patienten Informationen zur Erkrankung bereitstellen (Abb. 2).

In der speziellen Bedarfsevaluation wurde 120 Erstdiagnosen von Kopf-Hals-Karzinomen pro Jahr, basierend auf Daten aus dem klinischen Tumorregister, angenommen. Zusätzlich befinden sich unter Annahme eines 50%igen 5‑Jahres-Überlebens von 1000 im Tumorregister eingeschlossenen Patienten etwa 500 Patienten in der Nachsorge. Um die Implementierung simpel zu gestalten, sollen ePRO zunächst nur in der Nachsorge Anwendung finden. Die Nachsorgeintervalle sind seit Jahren etabliert [25]. Eine Änderung des Routinebetriebs war nicht erforderlich. Die Nachsorge erfolgt in der onkologischen Spezialsprechstunde einmal wöchentlich an 52 Arbeitswochen mit 35 Terminen, d. h,. im Jahr können 1820 Nachsorgen durchgeführt werden (Abb. 2).

#### Implementierungsplanung

Es wurde eine Hybridimplementierung mithilfe der Software „Computer-based Health Evaluation System“ (CHES) [27] gewählt. CHES ist eine Software für die Verwaltung, Speicherung und Darstellung von ePRO in Verbindung mit klinischen Daten. Entwickelt von der Fa. Evaluation Software Development (ESD, Innsbruck, Österreich), als akademisches Spin-off der Medizinischen Universität Innsbruck, umfasst CHES Funktionen wie die Verwaltung von ePRO-Fragebögen, webbasierte Komplettierung und Datenimport/-export mit begrenzten Schnittstellen [27]. Die Tirol Kliniken GmbH, als Krankenhausträger auf Landesebene, finanziert die Implementierung mit CHES, um das strategische Unternehmensziel der 10-Jahres-Digitalisierung voranzutreiben.

Die begrenzte Schnittstelle zwischen CHES und KIS ermöglicht Klinikern die Datenanalyse innerhalb des KIS. Die Dateneingabe durch Patienten ist an vielen Endgeräten möglich. CHES ermöglicht den nachträglichen Einschluss neuer Patientengruppen oder klinischer Pfade, generiert automatische Erinnerungen zur Dateneingabe und kann schwellenwertbasierte Rückmeldungen an Kliniker generieren. CHES erfüllt die oberste Priorität der Benutzerfreundlichkeit [27].

Das Projektteam entschied sich für die Implementierung des EORTC-QLQ-C30 [22], des EORTC H&N43 [23] und der HNC-FIT Scale [24]. Insgesamt füllen Patienten mit Kopf-Hals-Karzinomen in ihrer Nachsorge insgesamt 12-mal 73 Fragen aus, was etwa 30 min pro Assessment in Anspruch nimmt [22, 23]. Die HNC-FIT Scale wird vom Kliniker im Rahmen der klinischen Untersuchung in durchschnittlich 77 s komplettiert [24]. Diese begrenzte Auswahl soll den klinischen Mehrwert in Relation zur Belastung für Patienten mit Kopf-Hals-Karzinomen setzen. Um personelle Ressourcen zu schonen, wurde vorläufig auf eine automatische Rückmeldefunktion verzichtet.

Der ePRO-Prototyp wurde im November 2023 fertiggestellt. Für die Prototypenevaluation wurden 25 Anwender, davon 15 Ärzte (9 HNO-Ärzte, 2 Onkologen, 2 Strahlentherapeuten und 2 Phoniater), 4 Pflegekräfte, 4 Office Manager und 2 Psychologen, eingeschlossen. Davon waren 11 weiblich, das mittlere Alter betrug 39,9 (± 9; 21–58) Jahre, die mittlere Berufserfahrung 12,3 (± 7,6; 1,0–30,0) Jahre. Die korrekten Interpretationsraten für Symptomen und Funktion, erhoben mittels EORTC-QLQ-C30 [22], lag bei 96,7 bzw. 95,7 %, die für Funktion, erhoben mittels HNC-FIT Scale [23], bei 95,1 % (Abb. 1). Details zur Prototypenevaluation sind in Tab. 1 dargestellt. Die Anmerkungen zum zusätzlichen Nutzen und zu zusätzlichen Problemen sind in Tab. 2 und die Verbesserungsvorschläge in Tab. 3 zusammengefasst.

**Tab. 1:** Erhobenen Daten der ePRO-Prototypenevaluation

	Mittelwert	Standardabweichung	Minimum	Maximum
*Interpretationszeit* (min)	6,3	± 2,3	2,2	10,7
*Korrekte Interpretation* (%)				
Symptome^1^	96,7	± 6,9	76,9	100,0
Funktion^1^	95,7	± 9,0	60,0	100,0
Funktion^2^	95,1	± 9,0	75,0	100,0
*Erfahrung mit ePRO*	5,5	± 3,2	0	10
*Benutzerfreundlichkeit*				
Patientensicht	8,1	± 1,6	3	10
Anwendersicht	8,6	± 1,1	6	10
*Zusätzlicher Nutzen*	8,1	± 1,9	3	10
*Zusätzliche Probleme*^*3*^	7,0	± 1,5	4	10

^1^ erhoben mittels EORTC-QLQ-C30 („European Organization for Research and Treatment of Cancer Quality of Life Questionnaire – 30 items“) [22]

^2^ erhoben mittels HNC-FIT Scale (Head and Neck Functional Integrity Scale) [23]

^3^ höhere Werte entsprechend weniger zusätzlichen Problemen

**Tab. 2:** Anmerkungen zum ePRO-Prototypen

	Anzahl der Probanden	Anteil (%)
*Zusätzlicher Nutzen*		
„Schnellere Anamnese“	18	72
„Übersichtlicher“	8	32
„Patientenzentriert“	5	20
„Digitalisierung“	4	16
„Daten langfristig vorhanden“	4	16
„Weniger Zettelwirtschaft“	4	16
„Weniger organisatorischer Aufwand“	2	8
„Evaluation der Therapieoptionen“	2	8
„Qualitätssicherung“	2	8
*Zusätzliche Probleme*		
„Fehlende Zeit und Motivation des Personals“	13	52
„Problematisch bei älteren Patienten“	5	20
„Fehlende Compliance bei Patienten mit Kopf-Hals-Karzinomen“	5	20
„Symptome werden übersehen“	3	12
„Finanzierung“	3	12
„Patienten ermüden nach 20 Fragen“	2	8
„Daten werden fehlen“	2	8
„Technische Probleme“	2	8
„Ungeklärte Zuständigkeiten“	2	8
„Profitiert nur die Software-Firma“	1	4
„Rechtliche Probleme“	1	4

**Tab. 3:** Verbesserungsvorschläge für den ePRO-Prototypen

	Anzahl der Probanden	Anteil (%)
„Schwellenwerte für HNC-FIT Scale hinterlegen“	8	32
„Zusätzliche Farben einführen“	7	28
„Skalenniveaus vereinheitlichen“	6	24
„Automatisches Feedback“	4	16
„Selbsthilfeanleitungen hinterlegen“	4	16
„Anwendung auf allen Endgeräten“	3	12
„Patiententermine innerhalb von CHES darstellen“	2	8
„Patienten grafische Darstellung anbieten“	2	8
„QR-Codes für Registrierung“	1	4
„Direkte Integration in KIS“	1	4

*CHES* „Computer-based Health Evaluation System“ (Fa. Evaluation Software Development, ESD, Innsbruck, Österreich); *HNC-FIT Scale *Head and Neck Functional Integrity Scale,* KIS* klinisches Informationssystem

## Diskussion

ePRO sind ein innovatives Instrument für eine fokussierte, patientenzentrierte Anamnese [1–6]. Sie fördern die Selbstwirksamkeit der Patienten, was zu einer Reduzierung von Notaufnahmen und Hospitalisierungen führen kann [7, 13]. Die Anzahl erfolgreicher ePRO-Implementierung in der Klinik ist begrenzt [14–16]. Diese Arbeit skizziert den ePRO-Implementierungsprozess exemplarisch an der HNO Innsbruck.

An der HNO Innsbruck wurden befinden wir uns aktuell in der Präimplementierungsphase. Erfolgreich umgesetzte Kernschritte, gemäß den Vorgaben des NHS und der EORTC [20, 21], waren die Bildung eine Projektteams, Definition von Champions, die Bedarfsevaluation, Implementierungsplanung, Identifikation innerklinischer Barrieren, die Prototypenentwicklung und Evaluation.

Die Identifikation innerklinischer Barrieren erfolgt simultan zur Implementierungsplanung. So wurde entschieden, vorläufig nur Patienten mit Kopf-Hals-Karzinomen in die Nachsorge einzuschließen und die Erhebungszeitpunkte mit den klinischen Nachsorgen zu synchronisieren. Die Hybridimplementierung von CHES ermöglichte die Datenabfrage im bekannten KIS. Neben Benutzerfreundlichkeit und technischer Flexibilität trugen Synergieeffekte der Digitalisierungsstrategie des Krankenhausträgers positiv zur Finanzierung bei. Die Auswahl der Fragebögen zielte auf eine gesunde Relation zwischen klinischem Mehrnutzen und Patientenbelastung ab. Um Personal zu schonen, wurde vorläufig auf eine automatische Rückmeldefunktion verzichtet.

Das Feedback zum Prototypen war positiv. Die Bearbeitungszeit von 6,3 min nach Einschulung wurde als vertretbar empfunden. Die korrekte Interpretation lag in allen Kategorien über 95 %, auch für Anwender, die nicht in die Dateninterpretation involviert sein werden. Die Erfahrung mit ePRO an der HNO Innsbruck lag bei 5,5 von 10. Die Benutzerfreundlichkeit aus Patienten- und Anwendersicht lag bei 8,1 bzw. 8,6, was darauf hindeutet, dass der Prototyp benutzerfreundlich ist. Der zusätzliche Nutzen durch die Implementierung wurde hoch eingeschätzt (Bewertung: 8,1), wobei schnelle Anamnese (72 %), Übersichtlichkeit (32 %) und Patientenzentrierung (20 %) als Hauptvorteile genannt wurden. Die zusätzlichen Probleme wurden mit 7,0 gering bewertet, wobei Personal, Zeit und Motivation (52 %) sowie Bedenken hinsichtlich der Benutzerfreundlichkeit bei Älteren (20 %) und Patienten mit Kopf-Hals-Karzinomen (20 %) im Vordergrund standen. Verbesserungsvorschläge, z. B. das Hinterlegen von Schwellenwerten für die HNC-FIT Scale [24], wurden von 32 % der Anwender gefordert.

Es bleibt abzuwarten, ob die in der Bedarfsevaluation definierten Ziele in der Postimplementierungsphase im vierten Quartal 2024 erreicht werden können. Daten zu Symptomen, Lebensqualität und Funktion sollten dann vorliegen. Bei einer erfolgreichen Implementierung ist die Projektausweitung und Etablierung von schwellenwertbasierter automatischer Rückmeldung geplant.

## Fazit für die Praxis

- Elektronisch erfasste Patientenberichte („electronic patient-reported outcomes“, ePRO) bestehen hier aus digitalen, von Krebspatientenausgefüllten Fragebögen.
- Trotz Hinweisen auf eine Verbesserung der klinischen Versorgung ist die Integration von ePRO in der klinischen Kopf-Hals-Onkologie selten.
- Die Implementierung erfolgt in Phasen: Präimplementierung, Implementierung und Postimplementierung.
- Durchgeführt wird die Implementierung durch ein Projektteam und klinische „Champions“.
- Hybridimplementierungslösungen (z. B. „Computer-based Health Evaluation System“, CHES; Fa. Evaluation Software Development, ESD, Innsbruck, Österreich) bestehen z. B. darin, dass die Dateneingabe webbasiert, die Datenauswertung in das klinische Informationssystem (KIS) integriert erfolgt (limitierte Schnittstelle).
- In der Universitätsklinik für Hals‑, Nasen- und Ohrenheilkunde der Medizinischen Universität Innsbruck (HNO Innsbruck) liegt der Fokus auf der Nachsorge, dazu werden Fragebögen wie EORTC-QLQ-C30 („European Organization for Research and Treatment of Cancer Quality of Life Questionnaire – 30 items“), HNC-FIT Scale und EORTC Head and Neck Cancer Module (EORTC H&N43) 12-mal über 5,5 Jahre von den Patienten ausgefüllt, das sind 73 Fragen bei etwa 30 min Bearbeitungszeit.
- Die Prototypenevaluation erfolgte durch 25 Anwender: Sie wurde als benutzerfreundlich aus Patientensicht (8,1 ± 1,6; 3–10) und aus Anwendersicht (8,6 ± 1,1; 6–10) eingestuft.
- Der Hauptvorteil von ePRO ist eine schnellere Anamnese (72 %), Hauptnachteile sind Fehlen von Personal, Zeit und Motivation (52 %).
- Start der Implementierung war im ersten Quartal (Q1) 2024.
- Die Evaluation der Zielerreichung erfolgt im vierten Quartal (Q4) 2024.

## Data Availability

Die in dieser Studie erhobenen Datensätze können auf begründete Anfrage beim Korrespondenzautor angefordert werden.

## Abkürzungen

CHES: Computer-based Health Evaluation System
EORTC: European Organization for Research and Treatment of Cancer
ePRO: Elektronisch erfasste Patientenberichte
ESD: Fa. Evaluation Software Development
HNC-FIT Scale: Head and Neck Functional Integrity Scale
HNO Innsbruck: Universitätsklinik für Hals‑, Nasen- und Ohrenheilkunde der Medizinischen Universität Innsbruck
KIS: Klinisches Informationssystem
NHS: National Health Service
